# Ultrasound and Heat Treatment and Its Potential to Reduce Fennel Allergenicity

**DOI:** 10.3390/foods14132251

**Published:** 2025-06-25

**Authors:** Gordana Maravić-Vlahoviček, Mirela Marić, Marija Badanjak Sabolović, Suzana Rimac Brnčić

**Affiliations:** 1 Faculty of Pharmacy and Biochemistry, University of Zagreb, A. Kovačića 1, 10 000 Zagreb, Croatia; 2 Faculty of Food Technology and Biotechnology, University of Zagreb, Pierottijeva 6, 10 000 Zagreb, Croatia; mirela.maric1308@gmail.com (M.M.); marija.badanjak.sabolovic@pbf.unizg.hr (M.B.S.)

**Keywords:** allergens, Western blot, SDS-PAGE, ELISA, fennel

## Abstract

Fennel (*Foeniculum vulgare*), a member of the Apiaceae family, is known for its culinary and medicinal uses, as well as its potential to cause allergic reactions. Thermal and nonthermal technologies are commonly applied during the production of fruit and vegetable-based products, which may contain allergenic proteins. Consequently, understanding how these processing treatments affect allergenicity is crucial for managing allergenic risks during manufacturing and for identifying technologies that can reduce the allergenic potential of the final products. Currently, there is limited information available on how thermal and ultrasonic processing methods affect the allergenic properties of fennel. The aim of this study was to investigate the effects of ultrasound and heat treatment on the in vitro immunoreactivity of protein extracts from fennel. After sonication and heat treatment, the protein extracts were analyzed by denaturing polyacrylamide gel electrophoresis, Western blot and enzyme-linked immunosorbent assay. All treatments altered the protein patterns of fennel and partially degraded proteins in the range of 15–80 kDa. In addition, all treatments reduced IgE binding, indicating lower allergenicity. Western blotting with IgE from fennel-allergic patients confirmed these effects. The ultrasound probe had the strongest effect, almost eliminating IgE reactivity for several allergens. Heat treatment reduced allergenicity by about 30%, while sonication showed a reduction of about 15% and lower. A larger sample size is needed to better understand the effects of these treatments and the differences in individual allergic responses.

## 1. Introduction

Fruits, vegetables and herbs are key components of a healthy diet, providing essential antioxidants, vitamins, minerals and fiber. Despite their benefits, they can trigger IgE-mediated allergic reactions due to certain proteins in the plants. These plant allergies, while often mild, are relatively common, affecting up to 4.3% of the population for fruits and 1.4% for vegetables [1]. The high rates of cross-reactivity with birch and other pollens play a significant role in these allergies, especially in Europe, where pollen allergies currently affect up to 40% of the population [2]. Birch produces the most allergenic tree pollen [3]. For patients with food allergies, the only current solution is complete avoidance of the allergenic food.

In recent years, the consumption of canned fruits and vegetables has increased due to their health benefits as well as the demands of busy lifestyles and lack of time. These factors have prompted consumers to look for convenient meals with a longer shelf life that help reduce food waste and ensure year-round availability [4]. The global canned fruits market is expected to grow from USD 12.91 billion in 2025 to USD 17.12 billion by 2030, at a CAGR of 5.81%. [5]. In Europe, the market for canned fruit and vegetables is forecast to grow by 1–2% per year in the long term. This growth coincides with ongoing research into the effects of food processing on allergenic proteins and their structures. Researchers are investigating how different processing methods can alter protein structures in ways that affect allergenicity. In determining the changes that occur in food proteins, factors such as the processing method, the conditions, the food matrix and the duration of processing play a crucial role. During processing, food proteins can undergo a number of transformations, including denaturation, aggregation, restructuring of disulfide bonds and the generation of new inter/intra molecular bonds. Interestingly, the exposure of proteins to thermal and non-thermal processing techniques can potentially reduce or mitigate allergic reactions. Sometimes they reduce allergenicity, while in other cases they may increase it or even create new allergens (neoallergens). This research has important implications for the food industry as it could lead to the development of products with lower allergenicity, which could expand the market for consumers with food allergies.

Plant protein allergenicity is variably affected by thermal processing, with changes in epitope structure depending on treatment conditions. Thermal processing may include baking, pasteurization, blanching, boiling, autoclaving, roasting, microwave heating, ohmic heating, infrared heating, radio frequency heating and frying [6]. Although thermal processing can effectively modify the allergenic properties of food, it loses its attractiveness if the desired reduction can only be achieved at high temperatures, which can affect the organoleptic properties and nutritional content of the food. Thermal processing commonly leads to the loss of secondary and tertiary structures and non-covalent interactions and potentially disrupts or forms new epitopes, affecting IgE binding. Severe heat treatment can reduce the allergenicity of certain proteins, like the apple allergen Mal d 3, but may not eliminate it entirely. According to Sancho et al. [7], mild heat treatment of native Mal d 3 at 90 °C for 20 min did not significantly impact its IgE-binding capacity or mediator release, likely due to reversible unfolding. However, more severe heat treatment at 100 °C for 2 h led to structural changes that affected the protein’s ability to refold properly, resulting in reduced IgE-binding potency. This reduction was attributed to potential modifications such as the loss of a disulfide bond, which impaired conformational epitopes but not linear ones, and the presence of unmodified molecules that retained full refolding capability. Cuadrado et al. [8] evaluated the impact of boiling and autoclaving on the IgE-binding capacity of lentil and chickpea proteins. Boiling reduced the immunoreactivity of lentil allergens over time, but several allergenic proteins remained detectable. Autoclaving at higher pressures (2.6 atm) caused a more significant reduction in IgE-binding proteins, though some heat-stable bands persisted. For chickpeas, boiling and autoclaving also decreased immunoreactivity, with the strongest reduction at extreme conditions (2.6 atm, 30 min). Overall, autoclaving had a severe impact on protein integrity and IgE reactivity, especially at the highest pressure and duration tested. The study by Beyer et al. [9] suggests that roasting lupin seeds could reduce the allergenic potential of lupin proteins; however, in vivo studies are needed to confirm this. The modifications of hazelnut, walnut, almond, chestnut, cashew, pistachio and peanut allergenicity after thermal processing have been widely investigated [10,11,12]. Upon boiling for 60 min (100 °C), the allergenicity of peanut and pistachio is not affected [13]. However, roasting hazelnuts at 140 °C for 40 min significantly reduces their allergenicity. Nevertheless, some allergic individuals may still experience clinical symptoms, as the effectiveness of allergen reduction depends on various factors, including the roasting temperature, duration and specific method of heat treatment [14]. Autoclaving at 138 °C for 15–30 min has been shown to reduce the immunoreactivity of peanut, almond and chestnut allergens [15].

Cuadrado et al. [16] investigated the allergenic properties of hazelnuts, pistachios and cashews after subjecting them to various processing procedures, including heat (boiling at 100 °C for 60 min or autoclaving at 138 °C and 256 kPa for 30 min), pressure (steaming at 7 bar up to 170 °C for 120 s, followed by a rapid pressure drop to 50 mbar) and enzymatic digestion using food-grade proteases. In vitro tests (Western blot and ELISA) and in vivo tests (skin-prick) were performed to evaluate allergenic reactivity. It was found that enzymatic hydrolysis with proteases reduced the allergenic potential of tree nuts, and the combination with pressurized heating further reduced their allergenic capacity. The most effective approach was to use dynamic thermal high-pressure sterilization (DIC) prior to enzymatic digestion, which significantly reduced or eliminated the allergenic properties of the tree nuts.

Non-thermal processing technologies, including pulsed light, high-pressure processing, pulsed light, cold plasma, pulsed electric field and ultrasound processing, can help to reduce the allergenicity of food proteins by altering conformational epitopes [17]. High-pressure processing (HPP) has been proposed as a potential method to reduce the immunoreactivity of foods, as it causes denaturation and aggregation of the treated proteins, which typically occur at pressures of 200–300 MPa [18]. Pulsed ultraviolet light with wavelengths from 200 nm to 1000 nm can change the conformation of food allergens and cause protein aggregation, possibly resulting in the loss of conformal epitopes. However, it can also lead to the formation of reactive neoepitopes through the reassociation of residual peptides, possibly increasing the allergenic potential of the food. Plasma is a partially ionized gas composed of reactive species that can interact with proteins to alter their conformation while the pulsed electric field uses electric field strengths of 0.1 to 80 kV/cm to treat food and modify protein structures, resulting in a change in their functional properties. Pulsed electric field disrupts the secondary and tertiary structures of proteins by ionizing or destroying electrostatic interactions [19]. Ultrasonic treatment is widely used in the food industry for applications such as extraction, homogenization, freezing, thawing, emulsification and preservation. High-intensity ultrasound (20–100 kHz) induces physical and chemical changes in food through cavitation, where collapsing bubbles create high temperatures (up to 5000 K) and pressures (up to 1000 atm) [20,21]. This process also alters protein structures, leading to changes in allergenic properties due to denaturation and modification of protein conformation. Ultrasound’s shear forces and micro-streaming disrupt bonds, causing alterations in solubility, hydrophobicity and molecular weight [22]. Ultrasound has the potential to be combined with other technologies to significantly reduce the allergenicity of food. It can be applied in three ways, namely before, during or after other treatments, with each treatment affecting allergenicity through different mechanisms [23]. Studies show significant reductions in allergenicity for food products like milk and shrimp, with shrimp allergenicity decreasing by up to 75% in ELISA assays. Li et al. [24] demonstrated that high-intensity ultrasound treatment (30 Hz, 800 W for 30–180 min) reduced the allergenicity of shrimp, with the reduction being directly proportional to the duration of treatment, and that the purified shrimp allergen showed a greater reduction in allergenicity than the whole-shrimp extract under the same ultrasound conditions. Ultrasound-assisted high-temperature pressure treatment (ultrasound 500 W followed by HTP 0.14 MPa and 121 °C for 15 min) significantly altered the structure of clam tropomyosin by modifying its secondary structure. These structural changes disrupted the allergenic epitopes, resulting in a 68.1% reduction in the allergenicity of tropomyosin in clams [25]. Ultrasound treatment (25 kHz, 400 W) of kiwifruit for up to 16 min reduced total protein solubility by 20%, while it increased in vitro digestibility by 62% and peptide content by three times. The intense shear stress disrupted cellular and protein structures, altering secondary structures by decreasing alpha-helices and increasing beta-sheets. Additionally, the IgE-binding capacity of the allergen Act d 2 was significantly inhibited, resulting in a 50% reduction in its content after the 16 min ultrasound treatment [26]. The potential mechanisms by which non-thermal technologies may reduce food allergenicity are still being examined and require further research.

Fennel (*Foeniculum vulgare*), a member of the Apiaceae family, is known for its culinary and medicinal uses, but reports of fennel allergy are relatively rare [27]. The primary allergen identified in fennel is Foe v 1, which belongs to the profilin protein family. Profilins are pan-allergens that are commonly found in various plants and are known for causing cross-reactivity, especially with pollen allergens. In sensitive individuals, exposure to Foe v 1 can trigger allergic reactions, often related to cross-reactivity with other common profilin allergens, such as those from birch pollen. The study by Pastorello et al. [28] aimed to correlate the severity of allergic symptoms between peach and fennel and identify fennel allergens, focusing on the role of rPru p 3 antibodies. In 148 patients with peach allergy, 58 had both symptoms and IgE antibodies positive for fennel. The findings showed a significant association between severe reactions to peach and fennel (*p* = 0.0009). The major fennel allergen identified was a 9 kDa lipid-transfer protein (LTP) cross-reactive with Pru p 3, along with a 15 kDa protein related to Bet v 1. The study concluded that fennel should be included in the LTP syndrome due to its significant allergenicity.

The main goal of this study was to investigate the effects of ultrasound and heat treatment on the in vitro immunoreactivity of fennel protein extracts, since up to our knowledge, no studies about the influence of thermal and ultrasound treatment on fennel allergenicity have been reported. The analysis was conducted using electrophoretic and immunochemical methods.

## 2. Materials and Methods

The protein extracts from fennel bulbs (*Foeniculum vulgare*, Apiaceae) were kindly provided by Prof. Marie Antoniette Ciardiello from the Institute of Biosciences and Bio-resources (IBBR)—National Research Council, Italy. The extracts were prepared according to the method of Pastorello et al. [28], with some modifications. Briefly, 50 g of plant tissue was homogenized by dissolving 25 mL of 2 M NaCl, 2 g of polyvinylpolypyrrolidone (Sigma, Steinheim, Germany), 0.820 mL of 0.12 M ethylenediaminetetraacetic acid (Sigma, St. Louis, MO, USA) and 2.2 g of L-ascorbic acid (Panreac, Barcelona, Spain) in 25 mL of water, and the pH was adjusted to 3.5 by adding NaOH. After mixing in an ice-water bath for one hour, the samples were centrifuged at 17,300× *g* for 45 min. Approximately 50 mL of the supernatant, representing the protein extract, was collected. Samples were then dialyzed against 2 mM NaCl and concentrated by ultrafiltration using Ultracel 3K Amicon Ultra filters (Millipore, Carrigtwohill, Ireland). Ultrafiltration was stopped as soon as the sediment settled to the bottom of the tube. Protein concentrations were determined by the spectrophotometric Bradford method using bovine serum albumin (Carl Roth, Karlsruhe, Germany) as a standard for the calibration curve.

**Patients’ sera.**

Sera were obtained from four patients (three female and one male) suffering from adverse reactions to raw fennel. Human serum samples were selected based on the determination of specific IgE antibodies by a FABER test, which indicated high levels (≥0.30 FIU/mL) for fennel. Selected patients participated in the study based on ethics committee approval, and signed an informed consent form to ensure confidentiality and the protection of personal data. A single blood sample (15 mL) was taken, yielding blood serum containing IgE antibodies, which were used for further analysis. This study posed no risk beyond the routine risk associated with blood sampling. Participation in this study was entirely voluntary and informed consent for participation was obtained from all subjects involved in the study. The study was conducted in accordance with the Declaration of Helsinki, and the protocol was approved by the Ethics Committee of University of Zagreb School of Medicine (380-59/12-302/167) on 21 June 2012.

**Heat and ultrasound treatment**

Protein extracts from fennel bulbs at a concentration of 2 mg/mL were subjected to ultrasound and heat treatment. Two ultrasonic treatment methods were used to modify the protein extract. The first treatment was carried out in an ultrasonic bath (Elmasonic P300H, Elma, Germany) with eight ultrasonic probes acting on the samples in the bath at a frequency of 37 kHz, a total power of 380 W and an amplitude of 100%. The second ultrasonic treatment was carried out with an ultrasonic processor (QSONICA, Newtown, CT, USA) at a frequency of 20 kHz, a total power of 700 W and an amplitude of 100%.

A 1 mL portion of the protein extract was transferred into a 1.5 mL Eppendorf tube, and the samples were processed under the following conditions:

- Ultrasonic bath: 60 min at 40 °C
- Ultrasonic processor: 15 min at 60 °C
- Heat treatment: in a water bath at 95 °C for 1 min

After processing, the samples were stored at −20 °C.

**SDS–Polyacrylamide Gel Electrophoresis, SDS-PAGE**

Fennel protein extracts (10 µg protein per well) were separated in a gradient 8–16% denaturing polyacrylamide gel (Mini-PROTEAN^®^ TGX™ Precast Protein Gels, Biorad, Hercules, CA, USA) according to the manufacturer’s instructions [29]. To determine the position of the protein bands in the gel, the gel was stained with Coomassie blue stain and destained with 10% acetic acid in water [30]. The gel was analyzed and documented with an Amersham Imager 600 (GE Healthcare Life Sciences, Uppsala, Sweden). Analysis was performed in three independent experiments.

**Western blot**

Proteins from fennel extracts were separated by denaturing polyacrylamide gel electrophoresis, as described above. After electrophoresis, the gel proteins were transferred onto an Immobilon PVDF membrane (Sigma) using a semi-dry electrophoretic transfer device (The W.E.P. Company, Semi-Dry Blotting System, IMMTM-1-A, Saint Joseph, MI, USA) for 1 h at an electric current density of 0.8 mA/cm^2^. After overnight incubation at 4 °C in 3% BSA in TBS (0.5 M Tris/HCl, 1.5 M NaCl; pH 7.5), the membrane was washed in a TBST buffer (PBS, 0.1% Tween 20; pH 7.5) 3 times for 10 min. Then, the membrane was incubated for 2 h at 37 °C in the pooled serum of four subjects diluted in a ratio of 1:10 with the TBS buffer, and then washed in the TBST buffer 3 times for 10 min. The membrane was then incubated for 1 h in a 1:2000 detection antibody solution (goat IgG antibody against the human IgE molecule, conjugated with horseradish peroxidase, Sigma). Protein detection on the membrane was performed by the chemiluminescence method using the Amersham™ ECL™ Prime Western Blot Detection Reagent kit (GE Healthcare Life Sciences) according to the manufacturer’s instructions. The membrane was analyzed and documented using an Amersham Imager 600 (GE Healthcare Life Sciences). The analysis was performed in three independent experiments.

**Enzyme-linked immunosorbent assay—ELISA**

Fennel protein extracts were applied in duplicate to the wells of a microtiter plate in a volume of 100 µL (5 µg of protein per well with prior dilution of the extract with 50 mM carbonate buffer, pH 9.6 to a concentration of 50 µg/mL) and incubated overnight at 4 °C. The wells were washed 3 times with the PBST buffer (10 mM Na_2_HPO_4_, 137 mM NaCl, 2.7 mM KCl, 0.05% Tween^®^ 20; pH 7.4), followed by incubation in 200 µL of a 3% BSA solution in PBS for 1 h at 37 °C. After washing 3 times with PBST buffer, 100 µL of pooled serum from four subjects diluted 1:10 with PBS buffer (10 mM Na_2_HPO_4_, 137 mM NaCl, 2.7 mM KCl; pH 7.4) was added to the wells and incubated for 2 h at 37 °C. After washing with the PBST buffer 3 times, 100 µL of the detection antibody diluted with PBS buffer 1:2000 (goat IgG antibody against the human IgE molecule, conjugated with horseradish peroxidase, Sigma) was added to the wells, and incubated for 1 h at 37 °C. After washing with PBST 3 times, 100 µL of the substrate solution for horseradish peroxidase was added and the microtiter plate was incubated for 20 min at room temperature. The substrate o-phenylenediamine dihydrochloride (OPD) was dissolved to a final concentration of 0.5 mg/mL in the OPD buffer (0.05 M citric acid, 0.05 M sodium phosphate, pH 5.0) with the addition of 30% H_2_O_2_. The reaction was stopped by the addition of 2.5 M H_2_SO_4_. The absorbance at 490 nm was measured with a Wallac 1420 Victor2TM microtiter plate reader (Perkin Elmer Inc., Waltham, MA, USA). In the blind test, the protein extract was replaced with 50 mM carbonate buffer, pH 9.6, and the serum of a person who is not allergic to the fennel served as a negative control. The analysis was performed in three independent experiments.

**Statistical analysis**

Considering the small sample size, we used the nonparametric statistical analysis. Relative absorbance fractions were normalized using a Rankit transformation, which maps data ranks to standard normal quantiles [31]. A linear mixed-effects model was fitted to the transformed data, with treatment as a fixed effect and subject ID as a random intercept. A pseudo-control group (treatment = “NT”, relative absorbance fraction = 1) was included for baseline comparison using Dunnett’s post hoc test, with *p* < 0.0001 considered as a significant change [32]. We used R version 4.4.2 [33] and the following R packages: emmeans v. 1.10.7 [34] and lme4 v. 1.1.35 [35].

## 3. Results and Discussion

### 3.1. Electrophoretic Analysis of Protein Extracts

**Figure 1:**
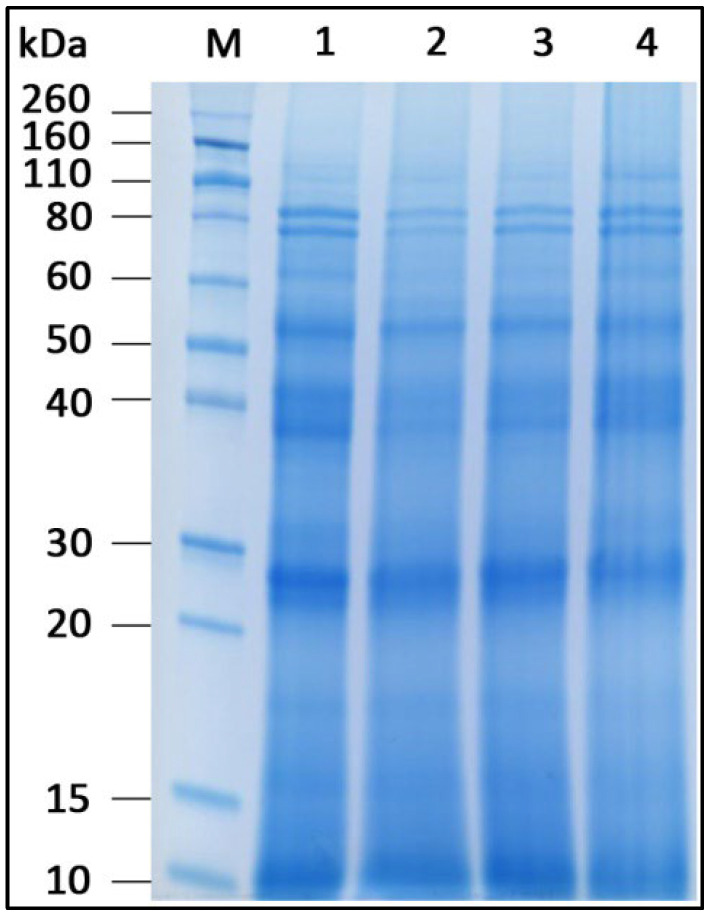
Electrophoresis of fennel protein extract. M: molecular mass standard; 1: untreated protein extract; 2: protein extract treated with an ultrasonic bath; 3: protein extract treated with an ultrasound probe; 4: protein extract exposed to a temperature of 95 °C.

To analyze the influence of different treatments on protein stability, protein extracts were subjected to denaturing polyacrylamide gel electrophoresis after treatment, as shown in Figure 1.

All three treatments change the fennel protein pattern compared to the untreated sample. The ultrasonic bath, similar to the treatment with the ultrasonic probe, leads to the partial degradation of several proteins with approximate molecular masses of 80 kDa, 75 kDa, 60 kDa, 42 kDa, 38 kDa, 17 kDa and 15 kDa, as indicated by the lower intensity of corresponding bands in the gel. The treatment with an elevated temperature of 95 °C affects all proteins equally, causing partial denaturation and subsequent loss of sharpness of all protein bands that appear smeared in the gel.

### 3.2. Immunochemical Detection of Allergens by Western Blot

**Figure 2:**
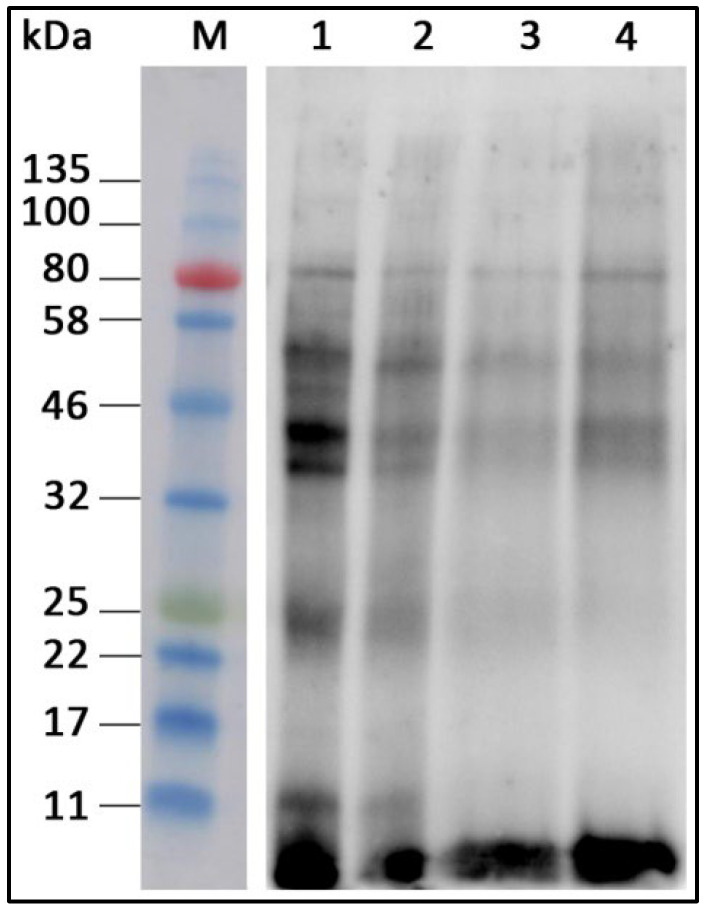
Western blot detection of fennel proteins. M: molecular mass standard; 1: untreated protein extract; 2: protein extract treated with an ultrasonic bath; 3: protein extract treated with an ultrasound probe; 4: protein extract exposed to a temperature of 95 °C.

The effects of different treatments on the allergenicity of individual proteins from fennel extracts were analyzed by Western blotting, using immunoglobulin E from the pooled serum of four allergic subjects that formed a complex with protein antigens in the extracts. The interaction was visualized by the binding of the detection antibody with the addition of the appropriate substrate Figure 2.

In the untreated protein extract, we identified several allergenic proteins with a wide range of molecular masses, from smaller ones of approximately 9, 11 and 24 kDa, to several proteins in the range from cca. 35 to 80 kDa. A small protein with an approximate molecular mass of 9 kDa and a slightly larger protein of cca. 40 kDa stand out as particularly strong allergens. Pastorello et al. [28] identified a 9 kDa protein as the main antigen in fennel and showed that it corresponds to the LTP (lipid-transfer protein). Lipid-transfer proteins (LTPs), also known as nonspecific lipid-transfer proteins (nsLTPs), are recognized for their ability to bind and transport hydrophobic ligands [36]. Although the full biological function of nsLTPs is not yet fully understood, these proteins are primarily involved in the transport of lipids between biological membranes and play a key role in defense, structural adaptation, antimicrobial activity and resistance to pathogens [37]. The lipid-transfer protein (LTP) is a major pan-allergen in *Rosaceae* and other plant foods, frequently causing cross-reactive allergic reactions in areas with low birch pollen prevalence, and has been detected in various foods like nuts, beer, maize, mustard and many fruits and vegetables, as well as in fennel [38]. Various heat treatments affect the allergenicity of plant protein families differently. While 2S albumins, nsLTPs, cereal prolamins, legumins, and vicilins are classified as heat-stable allergens, proteins from the PR-10 family are heat-labile [12]. Plant nsLTPs, known for their compact, predominantly helical structures stabilized by four disulfide bonds, exhibit strong resistance to heat and denaturation. Proteins such as these LTPs, which have additional intramolecular disulfide bonds, are not only more stable to thermal or acidic denaturation, but also tend to be more allergenic. A 15 kDa protein and several proteins in the 65–75 kDa range were detected as additional antigens [28]. In our study, the latter proteins were not detected as antigens, but this can be attributed to the differences among the subjects and the small number of subjects, as a consequence of the rare incidence of fennel allergy. All treatments reduced the allergenicity of the protein, resulting in the decreased intensity of individual bands. Treatment with an ultrasound probe had a particularly strong impact, where some allergenic proteins almost completely lost the ability to bind the IgE molecules from the serum of the allergic subjects.

### 3.3. Immunochemical Detection of Allergenic Proteins Using the ELISA Method

**Figure 3:**
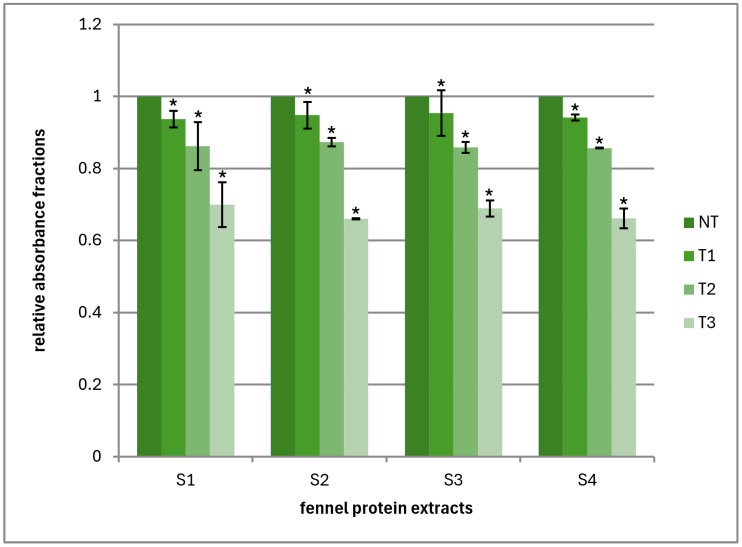
Relative absorbance fractions of treated fennel protein extracts versus the untreated sample. S1–S4—sera of four subjects allergic to fennel; NT—untreated protein extract; T1—protein extract treated with an ultrasonic bath; T2—protein extract treated with an ultrasound probe; T3—protein extract exposed to a temperature of 95 °C; Error bars ± SD (*n* = 3); *—treatments showing statistically significant reduction of A_490_ values (Dunnett’s post hoc test, *p* < 0.0001).

We examined the influence of different treatments on the allergenicity of total fennel proteins using the individual sera from four allergic subjects. The results comprise absorbance values at 490 nm after detection and visualization of the antigen–IgE antibody complex using a detection antibody conjugated with horseradish peroxidase and the corresponding substrate. The results are expressed as relative values of the proportion of treated samples against the untreated sample, which has an arbitrary value of one. The results are presented in the graphic form, where they are grouped together in relative ratios to observe the general impact of each treatment on the immunogenicity of the total proteins in the sample and to gain insight into the similarities and differences in the recognition of antigens by the sera of different subjects (Figure 3).

All treatments caused a decrease in the total immunogenicity of fennel proteins, with the most pronounced effect exhibited by the heat treatment, which resulted in a relative decrease of about 30%. This is followed by treatment with an ultrasound probe (reduction of about 15%) and treatment with an ultrasound bath, which had a very mild effect. We obtained similar results using Western blot, where the decrease in immunogenicity was most pronounced for the ultrasound probe treatment. It should be considered that ELISA detects the immunogenicity of total proteins, while Western blot distinguishes the effect of individual proteins, based on which we quantitatively assess the collective effect. This is not always accurate enough; therefore, the combination of both methods gives a more complete picture. Likewise, from the work of Pastorello et al. [28], we know that the main fennel antigen is LTP; thus, with different treatments, a small difference in the antigenicity of that single protein, which is difficult to see in a Western blot, can be differently reflected in the total protein immunogenicity measured by ELISA. The results also indicated that the sera of individual subjects reacted similarly with fennel proteins, but a stronger confirmation is needed from a study with a larger number of sera. The value of our results is also in the fact that the main antigens of fennel show cross-reactivity with peach antigens, so it is possible that different treatments can reduce the allergic reaction.

## 4. Conclusions

This study provides new insights into the potential of food processing methods—particularly ultrasound and heat treatment—to modify fennel proteins and reduce their allergenic potential. By demonstrating that all treatments altered protein patterns (in the range of 15–80 kDa) and reduced IgE binding, the results provide important evidence that such treatments can effectively reduce the allergenicity of fennel. Confirmation of these effects using sera from fennel-allergic individuals adds clinical relevance and strengthens the potential of the results. To date, the allergen-modifying effects of physical treatments of fennel proteins have been studied only to a limited extent. This study extends the current understanding by systematically comparing the efficacy of different treatments, focusing on the superior effect of ultrasound probe treatment in reducing IgE reactivity. The results suggest that ultrasound treatment could be a promising strategy to reduce the allergenicity of fennel, which in turn has implications for the development of hypoallergenic formulations for sensitive consumers. Furthermore, this approach could also be applied to other plant allergens. To build on these findings, future research should include larger and more diverse cohorts of allergic individuals to account for variability in IgE profiles and individual sensitivities and evaluate the combined effects of multiple treatments (e.g., heat + ultrasound) for synergistic allergen reduction.

## Data Availability

The original contributions presented in this study are included in the article. Further inquiries can be directed to the corresponding authors.

## Abbreviations

The following abbreviations are used in this manuscript:

SDS-PAGE	Sodium dodecyl sulfate–polyacrylamide gel electrophoresis
ELISA	Enzyme-linked immunosorbent assay
DIC	Dynamic thermal high-pressure sterilization
HPP	High-pressure processing
LTP	Lipid-transfer protein
